# The Granulocyte-colony stimulating factor has a dual role in neuronal and vascular plasticity

**DOI:** 10.3389/fcell.2015.00048

**Published:** 2015-08-07

**Authors:** Stephanie Wallner, Sebastian Peters, Claudia Pitzer, Herbert Resch, Ulrich Bogdahn, Armin Schneider

**Affiliations:** ^1^Department of Traumatology and Sports Injuries, Spinal Cord Injury and Tissue Regeneration Center Salzburg, Paracelsus Medical University SalzburgSalzburg, Austria; ^2^Department of Neurology, University Hospital RegensburgRegensburg, Germany; ^3^Interdisciplinary Neurobehavioral Core, Ruprecht-Karls-UniversityHeidelberg, Germany; ^4^University Clinic of Traumatology and Sports Injuries Salzburg, Paracelsus Medical University SalzburgSalzburg, Austria; ^5^SYGNIS Bioscience GmbHHeidelberg, Germany

**Keywords:** SCI, ALS, G-CSF, neurogenesis, arteriogenesis, angiogenesis, neuroregeneration, plasticity

## Abstract

Granulocyte-colony stimulating factor (G-CSF) is a growth factor that has originally been identified several decades ago as a hematopoietic factor required mainly for the generation of neutrophilic granulocytes, and is in clinical use for that. More recently, it has been discovered that G-CSF also plays a role in the brain as a growth factor for neurons and neural stem cells, and as a factor involved in the plasticity of the vasculature. We review and discuss these dual properties in view of the neuroregenerative potential of this growth factor.

## Introduction

In the last two decades the neuroscience community experienced a change of thought on the regeneration capacity of the central nervous system (CNS). The earlier dogma of incapability of CNS regeneration was overthrown by findings of astounding plasticity derived from neuron- or neuronal network- inherent adaptations, fostered by neurogenesis and by supporting vasculogenesis. These findings have raised hopes for developing new treatment approaches for neurological diseases.

Granulocyte-colony stimulating factor (G-CSF) is a 19.6-kDa glycoprotein which has been well-characterized as a growth factor for hematopoietic progenitor cells. It is an FDA- and EMA- approved drug commonly used to treat neutropenia and to mobilize bone marrow hematopoietic stem cells for transplantation (Nicola et al., 1983). For many years G-CSF has been thought of as a highly specific growth factor in the hematopoietic system. However, more recent studies have shown the presence of the G-CSF/G-CSF-receptor (G-CSFR) system in the brain and roles in neuroprotection, neural tissue repair as well as improvement in functional recovery have been described (Schneider et al., 2005b). The elevated expression of G-CSF/G-CSFR on neurons subjected to ischemia suggested that G-CSF serves as an autocrine protective signaling mechanism in response to neural injury. Furthermore, G-CSF receptors may also be present on glial cells, however at much lower levels (Schneider et al., 2005a). G-CSF exerts potent anti-apoptotic activity in neurons that appears largely responsible for its neuroprotective effects in acute injury models. In addition, G-CSF stimulates neurogenesis (Schneider et al., 2005b; Schmidt et al., 2015), arteriogenesis in the CNS (Sugiyama et al., 2011), and markedly improves long-term behavioral outcome after cortical ischemia (Schneider et al., 2005a) or spinal cord injury (SCI) (Dittgen et al., 2012). There is debate on the role of G-CSF-stimulated hematopoietic stem cells that may migrate to the injured CNS. Due to those effects and the ability of G-CSF to cross the intact blood brain barrier (Schneider et al., 2005b), facilitating peripheral administration, G-CSF got in the focus for treating acute and chronic neurodegenerative disorders, with protective and recovery enhancing effects in animal models of stroke, amyotrophic lateral sclerosis (ALS), Alzheimer's disease, Parkinson's disease, traumatic brain injury, and SCI (Diederich et al., 2012). In this review, we concentrate on direct and indirect effects of the cytokine G-CSF on CNS spinal regeneration especially by neurogenic and vasculogenic mechanisms and critically discuss the available pre-clinical and clinical evidence in SCI and ALS.

## G-CSF-mediated neural progenitor activation

The concept of adult neurogenesis is a relatively new one and has added one principally new option to influence plasticity and regeneration in the CNS. Neurogenesis can occur in the hippocampus (dentate gyrus), the olfactory bulb, and the subventricular zone (SVZ) (Winner and Winkler, 2015). There is debate whether adult neurogenesis occurs in the spinal cord near the central canal. One physiological role for hippocampal neurogenesis is pattern separation. There has been debate on whether neurogenesis is even relevant for humans, but detailed post-mortem studies on humans using radiocarbon dating in the brain have revealed that neurogenesis is particularly strong in the human hippocampus. Pathophysiologically, neurogenesis has been invoked as a possible causative or modifying factor in depression and schizophrenia. The case is stronger for depression, since several antidepressants stimulate neurogenesis (Hanson et al., 2011). Further, physical exercise which is anti-depressant, stimulates neurogenesis in rodents (van Praag et al., 1999; Yau et al., 2011) as well as humans (Déry et al., 2013). Interestingly, antidepressant medication for example fluoxetine, typically requires several weeks in order to be therapeutically active, which corresponds with the duration of newly generated nerves to be functional integrated. Moreover, impairment of neurogenesis has been shown to block the antidepressive activity of antidepressants such as fluoxetine (Santarelli et al., 2003). Also, imaging studies have shown a decrease in hippocampal volume in some cases. A function for G-CSF in stimulating neurogenesis was first described in 2005 in mice, treated subcutaneously with G-CSF (Schneider et al., 2005a). Neurogenesis was quantified using a standard bromodeoxyuridine (BrdU) incorporation protocol. While healthy mice showed a doubling of BrdU/neuronal nuclei (NeuN) double positive cells in the hippocampus 6 weeks after treatment initiation, mice subjected to cortical ischemia further increased that rate, pointing to a particular relevance of this stimulation also under pathological conditions to potentially enhance CNS plasticity. Most likely, stimulation of progenitor cells occurs via the G-CSF receptor present on these cells. Interestingly, the G-CSFR is already expressed at the embryonic stage in radial glia, which also later form the adult neural precursor cells (Kirsch et al., 2008). In G-CSF knock-out mice, hippocampal neurogenesis is strongly diminished, and the mice show deficits in behavioral plasticity (Diederich et al., 2009). For SCI and ALS the question whether neurogenesis occurs in the spinal cord and could be enhanced by G-CSF is of particular importance as this is the site of the main pathology. A careful review of the data on progenitor cells in the spinal cord comes to the conclusion that these cells exist in the ependymal zone of the central canal, but are restricted to a gliogenic fate (Sabelström et al., 2014). However, this restriction can be overcome by transplanting those cells into the hippocampus (Shihabuddin et al., 2000) and possibly growth factors like G-CSF could help to overcome this restriction in the spinal cord, but experimental proof for this is lacking so far.

## G-CSF-mediated vasculogenic effects

Prior to discussing effects on the vasculature it is important to clarify some conceptual issues. Overall, arteriogenesis refers to the remodeling of pre-existing capillary connections into conducting vessels by mechanisms involving shear stress and monocyte recruitment (Van Royen et al., 2001). Angiogenesis, on the other hand, describes the process of capillary sprouting driven by hypoxia and vascular endothelial growth factor (VEGF) (Heil et al., 2006). While angiogenesis is a key mechanism supporting tumor growth by improving local oxygen diffusion distances, it does not contribute to substantial increases in blood conductance, since only end-stage capillaries in the circulation are created (Buschmann and Schaper, 1999, 2000). Arteriogenesis is therefore a mechanism that is relevant for ischemic conditions such as vessel occlusions and decreased blood flow. The first hematopoietic factor for which evidence was found for a role in arteriogenesis was not G-CSF, but Granulocyte-macrophage colony stimulating factor (GM-CSF), as early as 2001 by Buschmann et al. (2001). GM-CSF was selected based on its propensity to stimulate monocyte and thrombocyte generation and the hypothesis of a key role of monocytes and thrombocytes in the arteriogenic process and stem cell niche. While these experiments made use of the femoral occlusion model in rabbits to observe arteriogenesis from pre-existing anastomosing capillaries, a publication from 2003 demonstrated also induction of arteriogenesis in the CNS (Buschmann et al., 2003). In this work, the authors made use of the 3-vessel occlusion (3-VO) model. In this model, both vertebral arteries and the left carotid artery are occluded. Treatment of rats over 7 or 21 days with 40 μg/kg/day GM-CSF led to a significantly larger increase in the diameter of the left PCA (posterior communicating artery) (72 vs. 39% increase in control animals after 7 days of treatment). Moreover, GM-CSF treatment also preserved good CO_2_-reactivity, indicating that the vessel was functionally intact with regard to blood flow regulation. Moreover, this treatment also resulted in an improved energy situation (ATP-depletion) in the hypoperfused brain in this 3-VO model (Schneeloch et al., 2004). In 2006, Deindl and colleagues were the first who also detect arteriogenic effects of G-CSF in a myocardial infarction model in mice. Treatment with 100 μg/kg/day G-CSF for 5 consecutive days after myocardial infarction led after 30 days observation to decreased mortality (68.8 vs. 46.2%), reduced final infarct size, and reduced thinning of the left ventricular wall. Moreover, hemodynamic parameters were improved such as ejection fraction, contractility, and relaxation of the ventricle. The authors could show G-CSF induced arteriogenesis from collateral vessels with proliferation of endothelial cells and smooth muscle cells (Deindl et al., 2006). Also, a decreased potential for arrhythmia generation in the infarcted heart was noted (Kuhlmann et al., 2006). A number of smaller clinical trials have indeed been conducted in patients with myocardial infarction with mixed results, the latest by Hibbert and colleagues in 86 patients (Hibbert et al., 2014). Later, arteriogenic effects of G-CSF were also noted in cerebral ischemia models (Sugiyama et al., 2011) and in the 3-VO model described above (Duelsner et al., 2012). G-CSF showed a very similar profile to GM-CSF in the 3-VO model in terms of doses used and treatment effects. In addition to the beneficial effects of arteriogenesis for blood flow enhancement, locally increased angiogenesis in the CNS could provide the critical neurovascular niche for neurogenesis and neuronal remodeling. Indeed, a number of articles suggest angiogenic activity of G-CSF in the brain. Bussolino and colleagues were the first to describe angiogenesis evoked by G-CSF (Bussolino et al., 1991). They used G-CSF secreting pellets implanted into the cornea of rabbits and observed formation of new capillaries within 8 days after implantation. Boiled G-CSF did not achieve this effect. *In vitro*, G-CSF enhanced proliferation and migratory behavior of HUVEC cells. In the brain, Lee et al. reported much later that the vascular surface area, vascular branch points, vascular length, number of BrdU-labeled endothelial cells, and endothelial nitric oxide synthase and angiopoietin 2 (ANGPT2) expression were all significantly increased in G-CSF–treated rats in the focal cerebral ischemia model (Lee et al., 2005). Ohki and colleagues found that G-CSF increased plasma levels of VEGF from neutrophils *in vivo* (Ohki et al., 2005). Furthermore, blockade of the VEGF pathway eliminated G-CSF–induced angiogenesis in a hindlimb ischemia model (measured as capillary density in the gastrocnemius muscle), suggesting that G-CSF–induced vasculogenesis is VEGF-dependent and could be indirectly mediated by this mechanism (Ohki et al., 2005). The finally responsible mechanism for these vasculogenic effects of G-CSF has not been unambiguously established. Direct effects on endothelial cells via the G-CSF receptor, increase in monocyte counts, and indirect effects via induction of VEGF release have all been discussed. In summary, G-CSF has both arteriogenic and angiogenic effects, thereby both enabling increased blood flow via generation of conductance vessels, and improving local oxygen diffusion and providing a neurovascular environment for repair mechanisms in the CNS.

## G-CSF in SCI

Acute SCI is associated with a significant burden of illness. Worldwide the estimated range of the incidence of SCI lies between 15 and 30 per million inhabitants per year (Wyndaele and Wyndaele, 2006). Therapeutic approaches in the acute phase including early resuscitation, steroid application, decompression/stabilization, have been reported to be associated with somewhat better outcomes in incomplete SCI. Thus, far, there is still no really effective treatment for SCI available, and the degree of neurological recovery is largely dependent on the magnitude of the initial injury. A considerable number of animal studies using various models of SCI have demonstrated convincing beneficial effects of G-CSF therapy (Table 1). Mechanisms triggered by G-CSF include anti-apoptotic effects and improved neurite outgrowth (Pitzer et al., 2010). Vasculogenesis certainly has a beneficial role for SCI repair and in limiting secondary damage after the initial traumatic event (Oudega, 2012). The degree of angiogenesis during the subacute phase after SCI correlates with regenerative responses, and the newly formed vascular bridge might provide scaffolding to accelerate axonal regeneration across the injury site. Angiogenesis might contribute to the regenerative response of neural tissue and enhance recovery of locomotor function after injury. Recent reports demonstrated the pro-regenerative effects of G-CSF in SCI which could be due to the enhancement of angiogenesis (Kawabe et al., 2011; Dela Peña et al., 2015). Kawabe and colleagues report significantly increased vessel numbers and increased expression of angiogenic cytokines after treatment with G-CSF in an experimental SCI model (Kawabe et al., 2011). G-CSF significantly promoted angiogenesis in both the white and gray matter of the spinal cord after injury. The total number of vessels with a diameter >20 μm was significantly greater and expression of angiogenic cytokines was significantly higher than in the control group. The G-CSF-treated group showed greater recovery of hindlimb function than the control group. As for a contribution of neurogenesis to the beneficial effects of G-CSF treatment, the problem with restricted neurogenesis in the spinal cord applies here. However, for SCI there is a way around this restriction and this is by making use of stem cell transplantation. An example of this approach was provided by Pan et al. (2008). Neural stem cells (NSC) were embedded in fibrin glue in combination with or without G-CSF and were transplanted into the gap in the injured spinal cord. Higher expression levels of NeuN and MAP-2 and advanced regeneration according to the clinical motor score, motor evoked potential and conduction latency was observed in the group treated the fibrin glue, G-CSF and NSC compared to the group without G-CSF (Pan et al., 2008). Based on the considerable pre-clinical evidence a number of smaller clinical trials have been initiated (Table 2). Several of these clinical studies demonstrated a neurological and functional improvement in SCI patients treated with G-CSF (Takahashi et al., 2012; Derakhshanrad et al., 2013; Inada et al., 2014; Saberi et al., 2014). Sakuma et al examined patients with a compression myelopathy and observed reduced progression of myelopathy in all 15 treated patients. Neurological improvements in motor and sensory functions were obtained in all patients after the administration, although the degree of improvement differed among the patients (Sakuma et al., 2012).

**Table 1:** **Published preclinical studies in the use of G-CSF for SCI**.

**Drug/dosage**	**Application/treatment duration**	**Outcome**	**SCI model/recipients**	**References**
G-CSF 200 μg/kg/day ± BMDC (4 weeks prior SCI)	sc. injected immediately after injury for 5 consecutive days	• Increased number of neutrophil antigen-negative BMDC in the lesioned site 24 h after injury • Increased number of BMDC 6 weeks after injury • Most of the BMDC in the lesioned site were Mac−1+ • Spared white matter was significantly larger • Recovery of hindlimb function	Compression model at T8 level, C57BL/6 mice	Koda et al., 2007
G-CSF 200 μg/kg/day	sc. injected immediately after injury for 5 consecutive days	• G-CSF Receptor expression by neurons in the spinal cord • Prevented glutamate-induced neuronal death during the acute phase • Increased number of surviving neurons after chronic phase • Increased recovery of hindlimb motor function	Compression model at T7-T8 level, BALBc/Cr mice	Nishio et al., 2007
G-CSF 50 μg/kg compared to MPSS	Single dose, injected sc. immediately after injury	• Decreased MPO, LPO activity and MDA concentration in the first 24 h after SCI, which may reduce tissue damage • No protective effect on the organelles after trauma	Contusion model at level T7-T9, Wistar albino rats	Sanli et al., 2010
G-CSF 50 μg/kg/day	sc. injection, within the first 72 h after injury for 3 consecutive days	• G-CSF Receptor expression on microglia cells • Microglia recruitment to the injury site in the first 72 h • Inhibited expression of pro-inflammatory factors promoted the expression of neurotrophic factors • Affect activation status of microglia • Inhibited the expression of markers of M1 macrophage in BV2 microglial cell line *in vitro* • Promoted microglia to adopt M2 phenotype • NFκB was involved in G-CSF induced M2 polarization • IBA1+ cells within the lesion site after G-CSF treatment • Attenuated accumulation of IBA1+ cells in the gray and white matter • Increased cell viability of BV2 microglial cell line • Reduced expression of NFκB in BV2 microglial cell line	Hemisection model at level T9-T11, Kunming mice	Guo et al., 2013
G-CSF 50 μg/kg/day ± NSC in the lesion	sc. injected immediately after injury for 5 consecutive days	• Improved motor function (BBB score) after 8 and 12 weeks • High expression levels of neuronal markers • Increased expression of GFAP • Increased BrdU positive cells • Regenerating tissue bridging with NSC and G-CSF	Transection model at level T8–T9, Sprague-Dawley rats	Pan et al., 2008
G-CSF 50 μg/kg/day ± BMC, MSC	sc. injection on day 7 after SCI for 5 consecutive days	• Improved functional recovery (BBB score, plantar test) • Improved behavioral parameters • Increased cross sectional areas of the white matter	Compression model at T8–T9 level, Wistar rats	Urdzíkova et al., 2006
G-CSF 300 μg/kg/day ± SCF	sc. injection on day 11 after SCI for 10 consecutive days	• Improved recovery of hindlimb motor function (BBB score) • Increased number of intrinsic BrdU+ cells • Increased number of intrinsic F4/80+ cells • Proliferation of OPC was activated • Activation of intrinsic spinal cord cell proliferation • Increase in intrinsic microglia cells was observed in lesion • No effects of SCF or G-CSF on the migration of transplanted BMDC to the lesion sites	Static contusion model at level T11-T12, C57BL/6 mice	Osada et al., 2010
G-CSF iv. 60 μg/kg sc. 30 μg/kg/day	Single dose injected iv. immediately after injury (5 min after surgery) followed by continuous sc. application for 2 weeks	• G-CSF Receptor is upregulated in the CNS upon SCI • Counteracts apoptotic cell death in the injured spinal cord • Increased expression of the anti-apoptotic Bcl-XL gene • Promotes neurite outgrowth *in vitro*	Transection model at level T8–T9 (transected to 75%), C57BL/6 mice	Pitzer et al., 2010
		• Enhanced branching capacity of hippocampal neurons • Effects on both the CST and serotonergic tracts • Increased number of large motoneurons • Improved functional connectivity post-injury • Improved functional outcome • G-CSF overexpression in CNS correlated with an improved functional motor outcome		
G-CSF iv. 60 μg/kg sc. 30 μg/kg/	iv. injection immediately after injury, followed by continuous sc. application for 14 days	• Improved functional recovery (BBB score, gridwalk test, and swim test)	Contusion model level T9-T10, Wistar rats	Dittgen et al., 2012
G-CSF 15 μg/kg/day	iv. injection, 1 h after injury and daily for 5 consecutive days	• Increased number of vessels with diameters > 20 μm • Increased expression of angiogenic cytokine mRNA (VEGF, HGF, FGF 2) • Promotion of functional recoverypermeability	Contusion model at level T8-T9, Sprague-Dawley rats	Kawabe et al., 2011
G-CSF 15 μg/kg/day	iv. injection, 1 h after surgery and daily for 4 consecutive days	• G-CSF Receptor expression on neurons, astrocytes and oligodendrocytes in normal spinal cord • G-CSF Receptor expression on GFAP+ astrocytes and MOSP+ oligodendrocytes • Increased number of MOSP+ oligodendrocytes • Suppressed inflammatory cytokine expression 72 h after injury • Smaller percentage of apoptotic oligodendrocytes 72 h and 1 week after surgery • Larger number of MAP-2+ neurons • Normal appearing myelin was higher than in control group • Better myelin integrity and preservation • Decreased Iba-1+ cell number • Improved hindlimb recovery	Contusion model at level T8–T9, Sprague-Dawley rats	Kadota et al., 2012
G-CSF 20 μg/ml ± GM-CSF	Single dose, injected ip. immediately after SCI	• improved functional recovery (BBB score) • Reduced cavity sizes • Marginal white matter seemed to be more intact • Repressed GFAP expression 1 and 4 weeks after injury • Repressed CSPG expression • Maintenance of the integrity of axon fibers • Suppressive ED-1+ cells 3 days after injury	Compression model at level T9, Sprague-Dawley rats	Chung et al., 2014

*BBB, Basso, Beatti, and Bresnahan locomotor rating scale; BMC, bone marrow cells; BMDC, bone marrow derived cells; BV2, micro glia cell line; CNS, central nerve system; CSPG, chondroitin sulfate proteoglycan; MSC, mesenchymal stem cells; CST, corticospinal tract; FGF2, fibroblast growth factor 2; GFAP, Glial fibrillary acidic protein; GM-CSF, granulocyte-macrophage colony stimulating factor; HGF, hepatocyte growth factor; IP, intraperitoneal; IV, intravenous; LPO, lipidperoxidation; M1, Macrophage type 1; M2, Macrophage type 2; MAP-2, microtubule associated protein; MDA, Malondialdehyde; MOSP, myelin oligodendrocyte-specific protein; MPO, Myeloperoxidase; MPSS, Methylprednisolone sodium succinate; NSC, neuronal stem cells; OPC, oligodendrocyte precursor cell; SC, subcutaneous; SCF, stem cell factor; SCI, spinal cord injury; VEGF, vascular endothelial growth factor*.

**Table 2:** **Published clinical studies on the use of G-CSF for SCI treatment**.

**Drug/dosage**	**Application/treatment duration**	**Outcome**	**Pat. Nr**.	**Patient characteristics**	**Side effects**	**References**
G-CSF (a) 5 μg/kg/day + surgery (*n* = 5) or (b) 10 μg/kg/day + surgery (*n* = 10)	iv. injection for 5 consecutive days	• Suppressed the progression of myelopathy in all 15 patients • Neurological improvements in both motor and sensory function	15	Cervical myelopathy patients, JOA score decreased during recent 1-month period	No serious adverse events occurred during or after treatment	Sakuma et al., 2012
G-CSF (Gran®) (a) 5 μg/kg/day (*n* = 5) or (b) 10 μg/kg/day (*n* = 11)	iv. injection, within 48 h after injury for 5 consecutive days	• AIS grade increased by one step in 80% of patients treated like a) and 50% of patients treated like b) • Increase in AIS motor scores detected 1 day after G-CSF administration in 10 μg group • Improvement in light touch and pin prick scores in the 10 μg group	16	Traumatic SCI patients recruited within 48 h of the primary injury	No severe adverse effects after G-CSF injection	Takahashi et al., 2012
G-CSF (Gran®) 10 μg/kg/day (*n* = 17) control group (*n* = 24)	iv. injection, within 48 h after injury for 5 consecutive days	• AIS grade improvement at least 1 step in 88.2% in the G-CSF group and 33.3% in the control group • Increased AIS improvement within patients with incomplete paralysis • No differences in pinprick score • Significant differences maintained 1 year after treatment	41	Traumatic SCI patients recruited within 48 h of the primary injury	No serious adverse effects (One patient in the G-CSF group developed fever proved to be an urinary tract infection One patient developed mild hepatic dysfunction)	Inada et al., 2014
G-CSF Filgrastim (Neupogen®) 5 μg/kg/day	sc. injection for 5 days	• Upper extremity but not lower extremity motor scores improved • Improved light touch sensory scores • Pinprick sensory scores improved • Increase in SCIM III score • Improvements in bladder and bowel management	19	Patients with chronic motor complete spinal cord injury, at least 3 months of active rehabilitation, at least 3 months duration of SCI	Mild side effects such as bone pain, rash, fever, neuropathic pain, and spasticity, all of them resolved after 1 week	Derakhshanrad et al., 2013
Filgrastim (Neupogen®) 5 μg/kg/day	sc. injection for 7 consecutive days	• Motor incomplete patients had increased improvement in AIS motor score, light touch, and pinprick sensory scores compared to motor complete patients • Less improvement in SCIM III scores of motor incomplete patients compared to motor complete patients	74	Traumatic SCI of at least 6 months duration, with stable neurological status in the last 3 months, undergone at least 3 months of standard rehabilitation, 52 motor complete and 22 motor incomplete patients	Not mentioned	Saberi et al., 2014

*AIS, ASIA Impairment Scale; ASIA, American Spinal Injury Association; JOA, Japanese Orthopedic Association score; SCIM Spinal Cord Independence Measure*.

## G-CSF and ALS: preclinical and preliminary clinical research

ALS represents a progressive and fatal neurodegenerative disease affecting motor neurons with a typical median disease course of 2–5 years and a lifetime risk of 1:400 (Ingre et al., 2015). A large number of clinical trials have been undertaken for this terrible disease, but the only approved drug so far is riluzole with limited treatment effects (Miller et al., 2012). Preclinical studies demonstrated that chronic or repeated subcutaneous administrations of G-CSF exert positive effects on survival rate, disease progression and immune status of SOD1-G93 mice (for details see Table 3). Some reports describe more gender-specific effects in ALS mouse models with positive effects on survival rate and motor performance only present in male but not female mice (Naumenko et al., 2011). Mechanisms that have been described include suppression of neuronal apoptosis and oligodendrocyte cell death by regulating the expression of apoptosis related proteins (Nishio et al., 2007; Pitzer et al., 2008). Another claimed mechanism of action is via mobilizing bone marrow cells into the spinal cord (Koda et al., 2007), or enhancing the availability of circulating hematopoietic stem cells in neuronal lesion sites and their ability for neurogenesis and angiogenesis (Nishio et al., 2007), although this is a very debated field. Moreover, reducing demyelination and expression of inflammatory cytokines such as TNF-α and IL-1β could be contributing to improved outcome after G-CSF treatment (Koda et al., 2007; Nishio et al., 2007; Kawabe et al., 2011; Kadota et al., 2012).

**Table 3:** **Published preclinical studies in the use of G-CSF for ALS**.

**Drug/dosage**	**Application/treatment duration**	**Outcome**	**SCI Model**	**References**
Filgrastim 30 μg/kg/day	sc. injection for 8 weeks via osmotic minipumps, mice at 11 weeks of age	• Prolonged survival of SOD1 mice • Delayed disease onset • Flattened disease progression • Flattened loss of grip strength • Reduced muscle atrophy • Larger muscle diameter • Decreased fibrillations • Ameliorated loss of motor neurons • No effect on microglial (Iba1) and astroglial (GFAP) markers • Reduced decrease of oligodendroglial markers (PLP) • Trend toward increased pan-neuronal markers (NSE)	SOD1-G93A mice	Pitzer et al., 2008
Filgrastim 30 μg/kg/day	sc. injection for 4 weeks via osmotic minipumps, mice at 11 weeks of age	• Restored transcriptomic deregulations of SOD1 mice • Transcriptome close to presymptomatic SOD1 mice or wild type animals • Modulation of genes closely related to neuromuscular functions (CCR4-NOT, Prss12)	SOD1-G93A mice	Henriques et al., 2014
Filgrastim 100 μg/kg/day	Week 10 of age until death via single sc. injections for 5 consecutive days per week	• Increased survival of SOD1 mice • Higher amount of surviving α-motoneurons • Increased amount of large myelinated axons • Increased microglia recruitment around neurons • Splenomegaly	SOD1-G93A mice	Yamasaki et al., 2010
Filgrastim 200 μg/kg/day ± surgery	Week 12 of age, single sc. injections from 5 days before hypoglossal axotomy until po. day 20 or day 40	• Increased viability rate of hypoglosal neurons • Increased number of Iba1-positive microglia	SOD1-G93A mice	Yamasaki et al., 2010
Pegfilgrastim 300 μg/kg/week	Week 12–16 of age, until clinical end stage via sc. single injections	• Prolonged survival of SOD1 mice • More sustained motoric capacity • No effect on spinal cord neuronal survival • Attenuated astrogliosis and microgliosis in the spinal cord • Attenuated production of inflammatory cytokines in microglia and peripheral monocytes • Reduced severity of muscle denervation	SOD1-G93A mice	Pollari et al., 2011
Pegfilgrastim 300 μg/kg/week	Week 12 of age until scarification via single sc. injections once per week	• Gender specific alterations • Reduced levels of reactive oxygen species in males but not females • No effect on survival rate or motor performance in females	SOD1-G93A mice	Naumenko et al., 2011
Adeno associated virus upregulation of endogenous G-CSF expression	One viral im. or is. injection (bilaterally at the L1 level)	• Delayed body mass decrease • Delayed paresis • Increased survival of SOD1 mice • Delayed clinical end point • Improved neuromuscular junction integrity and enhanced motor axon regeneration following nerve crush injury	SOD1-G93A mice	Henriques et al., 2011

*Iba1, ionized calcium-binding adapter molecule; IM, intramuscular; IS, intraspinal; GFAP, glial fibrillary acidic protein; NSE, neuron specific enolase; PLP, proteolipid protein; PO, post operation; SC, subcutaneous*.

Several angiogenic key players may be related to ALS with VEGF being the first described factor to contribute to ALS (Oosthuyse et al., 2001) and angiogenin (ANG) recently identified as a second angiogenic element related to that disease (Gao and Xu, 2008). VEGF represents a pro-angiogenic factor with neuroprotective properties. *In vitro* as well as *in vivo* studies showed that VEGF promotes neuronal survival (Silverman et al., 1999; Jin et al., 2000a,b) and prolongs the life span of ALS animal models (Lambrechts et al., 2003; Storkebaum and Carmeliet, 2004; Storkebaum et al., 2004). Several clinical studies investigate the VEGF-system as a possible treatment option for patients suffering from ALS (Pronto-Laborinho et al., 2014). An enhanced angiogenesis might protect motoneurons from degradation by increasing neurovascular perfusion. Studies demonstrate a correlative reduction in the human umbilical vein endothelial cell proliferative and angiogenic activities, which may contribute to the induction of ALS (Crabtree et al., 2007; Wu et al., 2007). Therefore, G-CSF might act as potent medication for the treatment of ALS by activating pro-angiogenic systems.

With regard to neurogenesis as a possible mechanism there are no data available from animal models that demonstrate a direct regenerative effect of G-CSF treatment on the first or second motoneuron. However, hippocampal and SVZ neurogenesis are certainly triggered and enhanced under the chronic G-CSF treatment protocols used in the animal studies that show beneficial effects of G-CSF treatment (Pitzer et al., 2008; Henriques et al., 2011) and one could speculate that this could indirectly functionally ameliorate the ALS phenotype via enhanced motor learning. There are papers that report enhanced progenitor cell proliferation and migration in the ependymal zone of the central canal in the lumbar spinal cord of ALS mouse models (Chi et al., 2006a,b, 2007), but true neurogenesis has not been reported in these studies. Based on these pre-clinical findings several smaller studies in ALS patients have been initiated with promising outcomes (Table 4) and no severe adverse effects even following long-term administration (Grassinger et al., 2014; Khomenko et al., 2015) Some studies reported no beneficial effects of G-CSF for patients suffering from ALS (Nefussy et al., 2010; Chiò et al., 2011).

**Table 4:** **Published clinical studies on the use of G-CSF for ALS treatment**.

**Drug/dosage**	**Application/treatment duration**	**Outcome**	**Pat. Nr**.	**Patient characteristics**	**Side effects**	**References**
Filgrastim (Neupogen®) 300 μg/kg/day ± CD133+ stem cells	3 days via single sc. injections/implantation of isolated CD133+ stem cells into the motor cortex	• No difference in FVC • Significant improvement of the ALSFRS score	23	Patients with confirmed ALS according to the clinical and neurophysiological criteria; no familiar ALS patients present	Not mentioned	Martinez et al., 2009
Filgrastim 300 μg/kg/day ± CD133+ stem cells	3 days via single sc. injections/implantation of isolated CD133+ stem cells into the motor cortex	• Positive tendency toward disease stabilization	67	Patients with confirmed ALS according to El-Escorial clinical and neurophysiological criteria	No severe adverse effects, mild and transient adverse effects; Major adverse effects after catheter insertion and stem cell transplantation including headache, insomnia, generalized malaise, back pain, vomiting and abdominal pain; One patient died due to acute subdural hematoma which occurred after treatment for myocardial infarction	Martínez et al., 2012
Filgrastim 10 μg/kg/day	day 1–10 and 20–25 via single sc. injections	• No differences in clinical tests (JTT, ALSFRS score, z-scores) reduced decline in fractional anisotropy	10	Patients with confirmed ALS according to the clinical and neurophysiological criteria	Mild to moderate adverse effects including well-described side effect profile of G-CSF including headache, bone pain and malaise	Duning et al., 2011
Filgrastim 300–600 μg/day	5 days via single sc. injections, 6 days via single injections	• No significant changes in disease progression	8	5 patients with confirmed ALS according to the clinical and neurophysiological criteria and 3 patients with probable ALS	No adverse effects	Cashman et al., 2008
Filgrastim 2 μg/kg/day	5 days via single sc. injection	• Reduced decline of ALSFRS score during first 3 month after treatment • Reduced decline of CAMP amplitude during first 3 month after treatment • Faster change of the ALSFRS score in the second 3 months after treatment	13	Patients with diagnosis of definite or probable ALS with duration less than 3.5 years	Mild adverse effects with 2 patients developing symptoms of infection and 3 other patients having mild fever	Zhang et al., 2009
Ratiograstim 5 μg/kg/day up to 10 μg/kg/day	One/two sc. injections for 5 consecutive days every 4 weeks	• Longtime safety and feasibility with no major side effects up to 3 years • Sustained mobilization of CFC • Loss of immature progenitors following long-term administration	6	Patients with diagnosis of definite or probable ALS according to the El-Escorial clinical and neurophysiological criteria	Mild, treatment-related adverse effects including bone pain, muscle pain, headache, fever, dyspnea and pre-syncope	Grassinger et al., 2014
Lenograstim (Myelostim®) 2 × 5 μg/kg/day	4 days every 3rd month via single sc. injection	• No effects on the decline of the ALSFRS-R score • No modification of quality of life • Reduced serum/CSF levels of MCP-1 • Reduced CSF levels of IL-17 • Enhanced serum levels of IP-10	24	Patients with diagnosis of definite, probable or probable-laboratory-supported ALS and less than 12 months disease duration	Few and transitory adverse effects including flu-like symptoms, nausea and asthenia; 2 severe adverse with 1 patient developing hyper prolactinemia and 1 patient developing thrombosis but both seem to be unrelated to study drug	Chiò et al., 2011
Filgrastim (Neupogen®) 5 μg/kg/day	4 days every 3rd via single sc. injections	• Trend of slowing disease progression	19	Patients with diagnosis of definite or probable ALS according to the El-Escorial clinical and neurophysiological criteria	Mild drug-related adverse effects including bone and muscle pain; no major adverse effects	Nefussy et al., 2010
Combination of PDgrastim, filgrastim, 300 μg recombinant G-CSF, equal to 30,000,000 IU of filgrastim, mannitol and sodium acetate 5 μg/kg/q12 h	5 days via single sc. injection	• No effect on the monthly ALSFRS-R score decline • No effect on the change of the ALS questionnaire and manual muscle testing scores • No effect on the change of the nerve conduction velocity • Lower elevation of CD34+ cell counts in female compared to male patients • Trend toward a greater ALSFRS-R score reduction in female G-CSF-treated patients compared to male placebo-treated counterparts	40	Patients with diagnosis of definite or probable ALS according to the El-Escorial clinical and neurophysiological criteria	Not mentioned	Amirzagar et al., 2015

*FVC, forced vital capacity; ALSFRS, ALS Functional Rating Scale; ALSFRS-R, ALS Functional Rating Scale Revised; JTT, Jebsen Taylor Test; CAMP, compound muscle action potential; CFC, colony forming cells; CSF, cerebrospinal fluid; MCP-1, monocyte chemoattractant protein-1; IL-17, interleukin-17; IP-10, interferon-induced protein 10; IU, international unit; SC subcutaneous*.

## Conclusion and clinical implications

In this review we have described the neuroregenerative potential of the hematopoietic growth factor G-CSF for SCI and ALS with a focus on vasculogenic and neurogenic mechanisms of action. Apart from all other arguments in favor of G-CSF as a novel type of neuroprotective drug, particularly due to its multiple mechanisms of action, and broad preclinical proof of principle one major advantage of this protein is that we are dealing with a drug with a well-known pharmacological profile, and an excellent safety record. Appropriate intervention in SCI depends on the nature, extent, and duration of the disease state, as pathophysiology can dramatically evolve over time. The initial mechanical damage occurs immediately after SCI followed by a cascade of potentially harmful secondary events that include the formation of free radicals, detrimental inflammatory responses and death of neurons and glia. A drug counteracting the induction of the secondary damage and promoting neuroregeneration is for major importance in the treatment of patients suffering from acute or chronic CNS diseases. Acute SCI causes immediate mechanical damage to the microvasculature of the cord followed by a secondary injury to the vessels, this combination produces spinal cord ischemia. Thus, angiogenesis is critically important to reduce secondary damage to the spinal cord vasculature. G-CSF plays a major function in the induction of angiogenesis and arteriogenesis which may promote neuroregeneration via the induction of a regeneration-friendly environment. How endogenous neurogenesis can contribute to SCI regeneration is unclear at present, spinal cord neurogenesis does not appear to play a significant role—central neurogenesis may however well-contribute to recovery processes due to an increased level of plasticity for central “rewiring” of descending motor pathways. Local transplantation approaches together with G-CSF appear as an attractive way toward exploiting neurogenesis as a repair mechanism.

Clinical trials conducted so far look promising, and at least G-CSF treatment appears feasible and safe. However, proper controlled and randomized trials are lacking to draw sound conclusions. Many questions are on the table regarding the timing of treatment post-injury: (acute vs. delayed), G-CSF dosing (high dose, low dose), the treatment mode (chronic continuous vs. intermittent; intravenous vs. subcutaneous or even locally applied). For ALS, the antiapoptotic properties, protection of the neuromuscular junction, immune modulating, as well as angiogenic properties appear key in counteracting the pathology. As in SCI, a multitude of pathophysiological processes need to be addressed together, which is an argument for a growth factor as therapeutic agent (Henriques et al., 2010). The direct impact of the neurogenic potential of G-CSF for modifying the disease course is not so clear at the present state of knowledge. As is the situation with SCI, there are interesting clinical studies that indicate feasibility and safety of treatment, and show promising hints for efficacy, but larger randomized controlled studies are needed to get definite answers. Imaging (Duning et al., 2011) and potentially other biomarkers may help in clinical development in ALS. Several questions for clinical development remain open, in particular feasibility and safety of a potentially yearlong treatment. Very recent and promising data support this (Grassinger et al., 2014; Khomenko et al., 2015).

### Conflict of interest statement

The authors have read the journal's policy and have the following conflicts: Armin Schneider and Ulrich Bogdahn are inventors on patent applications claiming the use of G-CSF for spinal cord injury treatment. There are no other patents, products in development or marketed products to declare. This does not alter the authors' adherence to all the Frontiers policies on sharing data and materials. The other authors declare that the research was conducted in the absence of any commercial or financial relationships that could be construed as a potential conflict of interest.

## Abbreviations

ALS: amyotrophic lateral sclerosis
ANG: angiogenin
ANGPT2: angiopoietin 2
BrdU: bromodeoxyuridine
CNS: central nervous system
EMA: european medicines agency
FDA: food, and drug administration
G-CSF: Granulocyte-Colony Stimulating Factor
G-CSFR: Granulocyte-Colony Stimulating Factor Receptor
GM-CSF: Granulocyte-macrophage colony stimulating factor
HUVEC: human umbilical vein endothelial cells
IL: interleukin
kDa: kilo Dalton
MAP-2: microtuble-associated protein 2
NeuN: neuronal nuclei
NSC: neuronal stem cell
PCA: posterior communicating artery
SCI: Spinal Cord Injury
SOD1: superoxide dismutase 1
SVZ: subventricular zone
TNF: tumor necrosis factor
VEGF: vascular endothelial growth factor
3-VO: 3-vessel occlusion.
